# Early Amyloid Detection in Idiopathic Carpal Tunnel Syndrome: A Puzzling Gap Between Peripheral and Cardiac Involvement

**DOI:** 10.3390/jcm15176543

**Published:** 2026-08-24

**Authors:** Ana Martins, Raquel Machado, Sofia Pimenta, Janete Santos, Hugo Osório, Pedro Madureira, Francisco Serdoura, Elsa Fonseca, Barbara Pereira, Lúcia Costa, Elisabete Martins

**Affiliations:** 1 Department of Rheumatology, Centro Hospitalar Universitário de São João, 4200-319 Porto, Portugal; anaigmartins.med@gmail.com (A.M.);; 2 RISE-Health, Faculdade de Medicina, Universidade do Porto, 4200-319 Porto, Portugal; 3 E2S, Politécnico do Porto, 4200-072 Porto, Portugal; 4 Department of Medicine, Faculdade de Medicina, Universidade do Porto, 4200-319 Porto, Portugal; 5 i3S—Instituto de Investigação e Inovação em Saúde, Universidade do Porto, 4200-135 Porto, Portugal; 6 Ipatimup—Instituto de Patologia e Imunologia Molecular, Universidade do Porto, 4200-135 Porto, Portugal; 7 Department of Pathology, Faculdade de Medicina, Universidade do Porto, 4200-319 Porto, Portugal; 8 Department of Orthopaedics, Centro Hospitalar Universitário de São João, Porto, 4200-319 Porto, Portugal; 9 Pathology Service, Centro Hospitalar Universitário de São João, 4200-319 Porto, Portugal; 10 Department of Nuclear Medicine, Centro Hospitalar Universitário de São João, 4200-319 Porto, Portugal; 11 Department of Cardiology, Centro Hospitalar Universitário de São João, 4200-319 Porto, Portugal

**Keywords:** carpal tunnel syndrome, cardiac amyloidosis, tenosynovial biopsy, cardiac scintigraphy

## Abstract

**Background/Objectives**: Idiopathic Carpal Tunnel Syndrome (CTS) can be an early manifestation of systemic amyloidosis, particularly transthyretin cardiac amyloidosis (ATTR-CA). The primary purpose of this retrospective case series was to describe the presence of amyloid deposits in tenosynovial tissue and explore potential cardiac involvement in patients undergoing carpal tunnel release surgery. **Methods**: From a cohort of 54 patients diagnosed with bilateral idiopathic CTS with surgical indication, 12 patients were selected for tenosynovial tissue samples, which were subsequently evaluated using Congo red staining and proteomic confirmation via mass spectrometry. Before the procedure, patients underwent a clinical assessment of medical history, electrocardiogram, and cardiac scintigraphy with Technetium-99 m 3,3-diphosphono-1,2-propanodicarboxylic acid (^99m^Tc-DPD). Transthoracic echocardiogram and cardiac magnetic resonance were subsequently performed in all patients with positive scintigraphy, while a subset of scintigraphy-negative patients underwent an echocardiogram. **Results**: Congo red staining identified amyloid deposits in 3 of the 12 patients (25%). Proteomic analysis confirmed ATTR amyloidosis deposits in 2 of these patients (17%). One of these 2 patients presented Perugini grade 3 uptake on cardiac scintigraphy, suspicious for ATTR-CA. Complete concordance across histology, proteomics, and cardiac imaging was observed in only 1 patient (8.3%). Three discordances were noted: one case of tenosynovial ATTR without evident cardiac disease, one patient with a discordant Congo red result likely reflecting low amyloid burden or tissue heterogeneity, and one with imaging findings suspicious for ATTR-CA despite a negative tenosynovial biopsy. **Conclusions**: Tenosynovial biopsy obtained during CTS surgery can reveal early amyloid deposition, which may precede overt cardiac involvement. The variability observed across findings underscores the need for a multimodal diagnostic approach that integrates histological, proteomic, and imaging data, thereby mitigating the risk of amyloidosis misclassification.

## 1. Introduction

Carpal Tunnel Syndrome (CTS) is the most common type of entrapment neuropathy and affects approximately 1–5% of the general population, particularly patients older than 50 years [1]. CTS can be linked to various conditions and diseases, including pregnancy, obesity, diabetes, and amyloidosis [2]. Nonetheless, the exact cause of CTS remains unclear in most cases.

For over 60 years, hand surgeons and other physicians have observed the presence of amyloid, a tough and grayish-white substance composed of misfolded protein, in patients undergoing carpal tunnel release [3]. The prevalence of amyloid deposition in patients with CTS has been the subject of investigation in several studies, with reported rates ranging widely from 9 to 34% [2,4,5,6,7]. Histological and immunohistochemical analyses in these cohorts have consistently identified transthyretin (TTR) as the predominant amyloidogenic protein [5,6]. These cumulative findings suggest a close and robust association between the deposition of TTR and the onset of CTS.

Recent studies have shown that CTS can be an initial symptom of systemic ATTR amyloidosis, particularly ATTRwt [2,8,9] and have hypothesized it as an early symptom of ATTR cardiomyopathy [7]. Systemic ATTRwt is an aging-related disorder, usually associated with cardiac disease, but it can also affect other organs. This condition is rarely recognized before death, and at diagnosis, most patients present with advanced-stage disease, with few therapeutic options. Postmortem studies have identified wild-type TTR deposition in 12–25% of people older than 80 years and in 37% of people older than 95 years [2,3]. Among individuals older than 80 years, 25% have TTR deposits in the heart. Cardiac scintigraphy with Technetium-99 m 3,3-diphosphono-1,2-propanodicarboxylic acid (^99m^Tc-DPD) is a valuable diagnostic modality for cardiac amyloidosis (CA) as it demonstrates high sensitivity and specificity in detecting myocardial infiltration across a wide spectrum of cardiac involvement [10,11,12,13].

The CarPoS study (NCT05409833) is a cross-sectional clinical study aimed at providing additional data on the association between CTS and transthyretin cardiac amyloidosis (ATTR-CA). It has received ethical approval (reference No: CE 200/2020) and, as such, adheres to established guidelines. Therefore, the specific purpose of this retrospective case series was to describe the presence of amyloid deposits in tenosynovial tissue and characterize subsequent cardiac involvement in patients undergoing carpal tunnel release surgery using a multimodal diagnostic approach that integrates histological, proteomic, and imaging data.

## 2. Materials and Methods

The CarPoS study (NCT05409833) is a cross-sectional clinical study aimed at providing additional data on the association between CTS and ATTR-CA. Inclusion criteria were patients more than 60 years old with symptomatic bilateral idiopathic CTS and with surgical indication. Patients with diabetes, hypothyroidism, chronic renal failure under hemodialysis, inflammatory arthropathies, multiple myeloma, gout, chondrocalcinosis, Colles’s fracture, space-occupying lesions, and infectious synovitis were excluded. Between July 2020 and July 2026, a total of 54 patients were enrolled in the prospective cross-sectional CarPoS study. For this specific retrospective case series, 12 consecutive patients were selected from the broader CarPoS cohort based strictly on the availability of tenosynovial tissue samples collected during CTS surgery. Tissue availability for this sub-analysis was determined by the surgeon’s discretion to send tissue for histological analysis during the standard procedure.

The project has been approved by the Ethics Committee for Health of São João University Hospital (reference No: CE 200/2020), according to the principles of the Helsinki Declaration, the Convention on Human Rights and Biomedicine, and the guidelines of the Council for International Organizations of Medical Sciences, and written informed consent was obtained from each patient.

All patients with bilateral CTS, confirmed by electromyography (EMG), were submitted to medical history, clinical examination, laboratory work-up (including renal function, thyroid-stimulating hormone (TSH), rheumatoid factor (RF), and antinuclear antibodies (ANAs) to exclude secondary causes of CTS), wrist ultrasound, electrocardiogram (ECG), and cardiac scintigraphy with ^99m^Tc-DPD before the carpal tunnel release surgery. Because this study originated in a non-cardiac screening setting, the study protocol relied on ^99m^Tc-DPD scintigraphy as the primary screening tool for ATTR-CA. Consequently, transthoracic echocardiogram (TTE) and cardiac magnetic resonance (CMR) were systematically performed only in patients with positive scintigraphy. A subset of scintigraphy-negative patients underwent TTE, but this was performed at the discretion of their attending physicians based on independent clinical judgment, rather than as a mandated step in the study protocol.

Wrist ultrasound was performed by the same rheumatologist, who assessed the cross-sectional area of the median nerve at the tunnel inlet. The presence of synovitis, tenosynovitis and space-occupying lesions was also evaluated, with the specific aim of excluding secondary causes of CTS, if present, and ensuring the inclusion of idiopathic cases only. Nerve conduction studies, including motor and sensory assessments across the carpal tunnel, were conducted and classified based on severity, categorizing the involvement as mild, moderate, or severe. CTS diagnosis relied on a combination of clinical history, physical examination, nerve conduction studies, and wrist ultrasound. 

Cardiac scintigraphy was performed 2 h after the intravenous administration of 20 mCi of ^99m^Tc-DPD. The acquisition protocol included whole-body planar images, regional anterior, posterior, and lateral planar views of the thorax and wrists, and Single-Photon Emission Computed Tomography (SPECT) of the thoracic region. The integration of SPECT imaging was standard practice to accurately differentiate true myocardial uptake from intracavitary blood pool or adjacent rib uptake. Scans were evaluated, and the Perugini grading was assigned strictly on planar imaging by the same experienced nuclear medicine physician, who was completely blinded to the patients’ clinical, histological, and proteomic data. The integration of SPECT imaging was standard practice to accurately confirm myocardial localization and differentiate true myocardial uptake from intracavitary blood pool or adjacent rib uptake.

Tenosynovial tissue samples collected during carpal tunnel release surgery were routinely formalin-fixed and paraffin-embedded. Tissue sections were stained with Congo red and subsequently examined by experienced pathologists using both bright-field and cross-polarized light microscopy to identify the characteristic apple-green birefringence indicative of amyloid deposition. Because this initial histological evaluation was performed as part of the standard diagnostic workflow, the pathologists were completely blinded to the subsequent proteomic and scintigraphic screening results.

Whole tenosynovial tissue samples were processed for proteomic analysis without laser microdissection, following the solid-phase-enhanced sample-preparation (SP3) protocol and trypsin/LysC digestion [14]. Protein identification and label-free quantitation were performed via nanoLC-MS/MS using a Vanquish Neo liquid chromatography system coupled to an Eclipse Tribrid Orbitrap mass spectrometer with a FAIMS interface (Thermo Scientific, Waltham, MA, USA). Raw data were processed using Proteome Discoverer 3.1.1.93 and searched against the UniProt *Homo sapiens* database (2024_01), applying a strict target false discovery rate (FDR) of 1% at the peptide and protein levels. Amyloid typing was determined by the relative abundance of specific amyloidogenic proteins (e.g., TTR) alongside canonical amyloid signature proteins, such as Apolipoprotein E (APOE) and Serum amyloid P-component (APCS). Operationally, samples were classified as compatible with ATTR deposition when they demonstrated a markedly elevated normalized abundance of these key proteins compared to the baseline expression of the negative cohort. Detailed quantitative proteomic data, specifically the normalized protein abundances used for this comparative analysis, are provided in Supplementary Table S1.

## 3. Results

A total of 12 patients underwent tenosynovial biopsy. The median age was 65 years, ranging from 60 to 86, and 75.0% (n = 9) were female. All 12 included patients had normal renal and thyroid function, and negative screening for inflammatory arthropathies (including negative RF and ANAs, confirming the idiopathic nature of their CTS.

Table 1 summarizes the clinical characteristics of the study population. Only one patient had previous history of cardiovascular disease (Case #1), while hypertension and dyslipidemia were present in 50.0% (n = 6) and 58.3% (n = 7) of patients, respectively.

Detailed cardiologic characteristics, including echocardiographic, cardiac magnetic resonance, and biomarker profiles, are summarized in Table 2.

**Figure A1:**
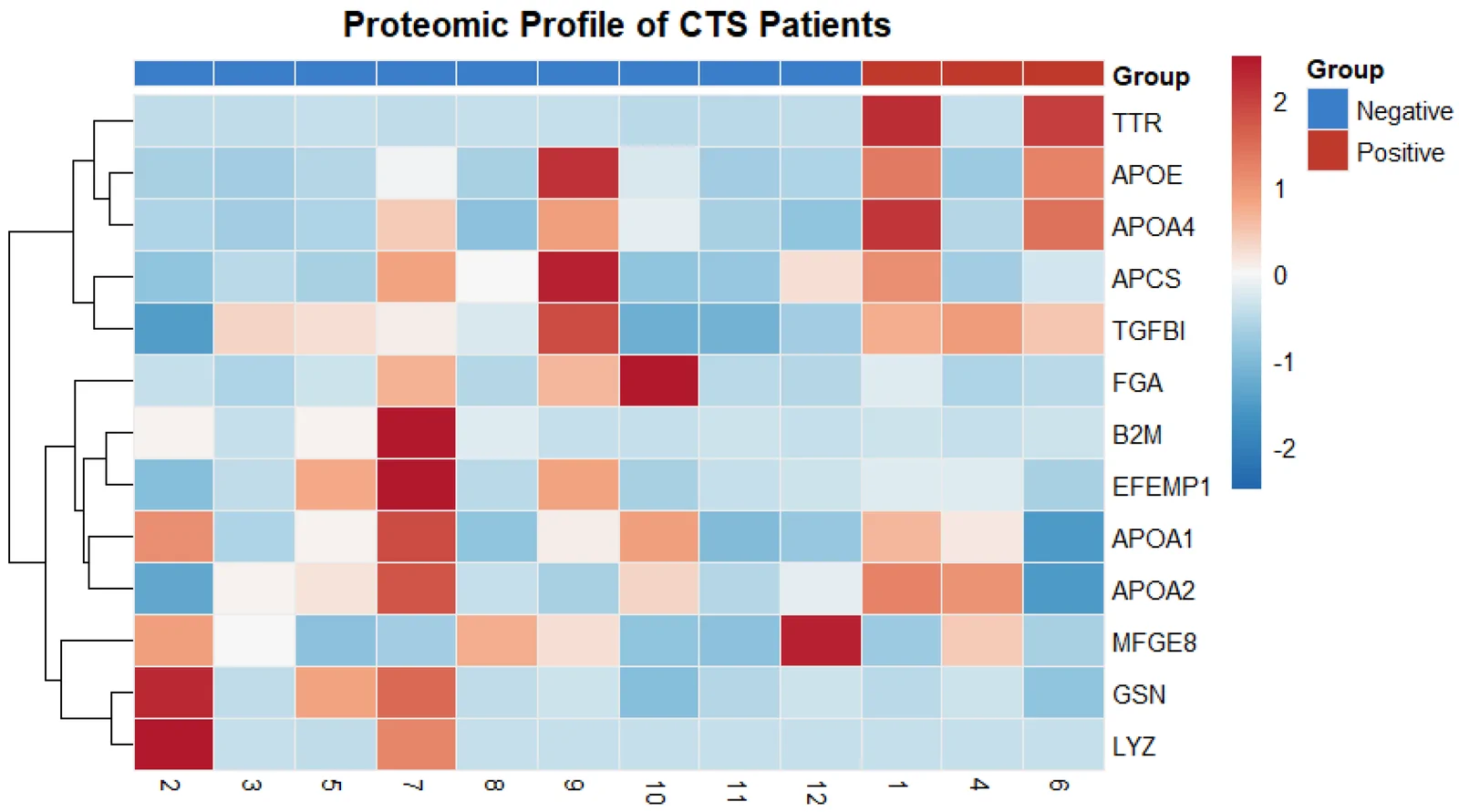
Proteomic heatmap of tenosynovial tissue samples from patients with carpal tunnel syndrome (CTS). The colour scale represents row-scaled normalised protein abundances (Z-scores), ranging from low (blue) to high (red) relative expression. Normalisation was based on the total peptide amount. Proteins (rows) were grouped using hierarchical clustering, as indicated by the dendrogram on the left. Patients (columns) were deliberately ordered and grouped by their Congo red staining results (Negative vs. Positive) to facilitate visual comparison. Any missing quantitative values were natively handled during the initial data export from the Proteome Discoverer pipeline prior to our downstream comparative analysis. The displayed proteins were specifically selected based on their known roles as amyloidogenic drivers or canonical amyloid-signature proteins (e.g., TTR, APOE, APCS, B2M). This descriptive heatmap is supported by the formal quantitative proteomic data provided in Supplementary Table S1.

Congo red staining identified amyloid deposits in 3 of the 12 patients (25%, 95%CI [5, 57]): Cases #1, #4, and #6. Among these, proteomics analysis confirmed ATTR amyloid deposition in two patients, Cases #1 and #6 (17%, [2, 48]) [Figure A1]. In addition, two patients, Cases #6 and #7, showed Perugini grade 3 uptake on cardiac scintigraphy, highly suspicious for ATTR-CA.

**Figure 1:**
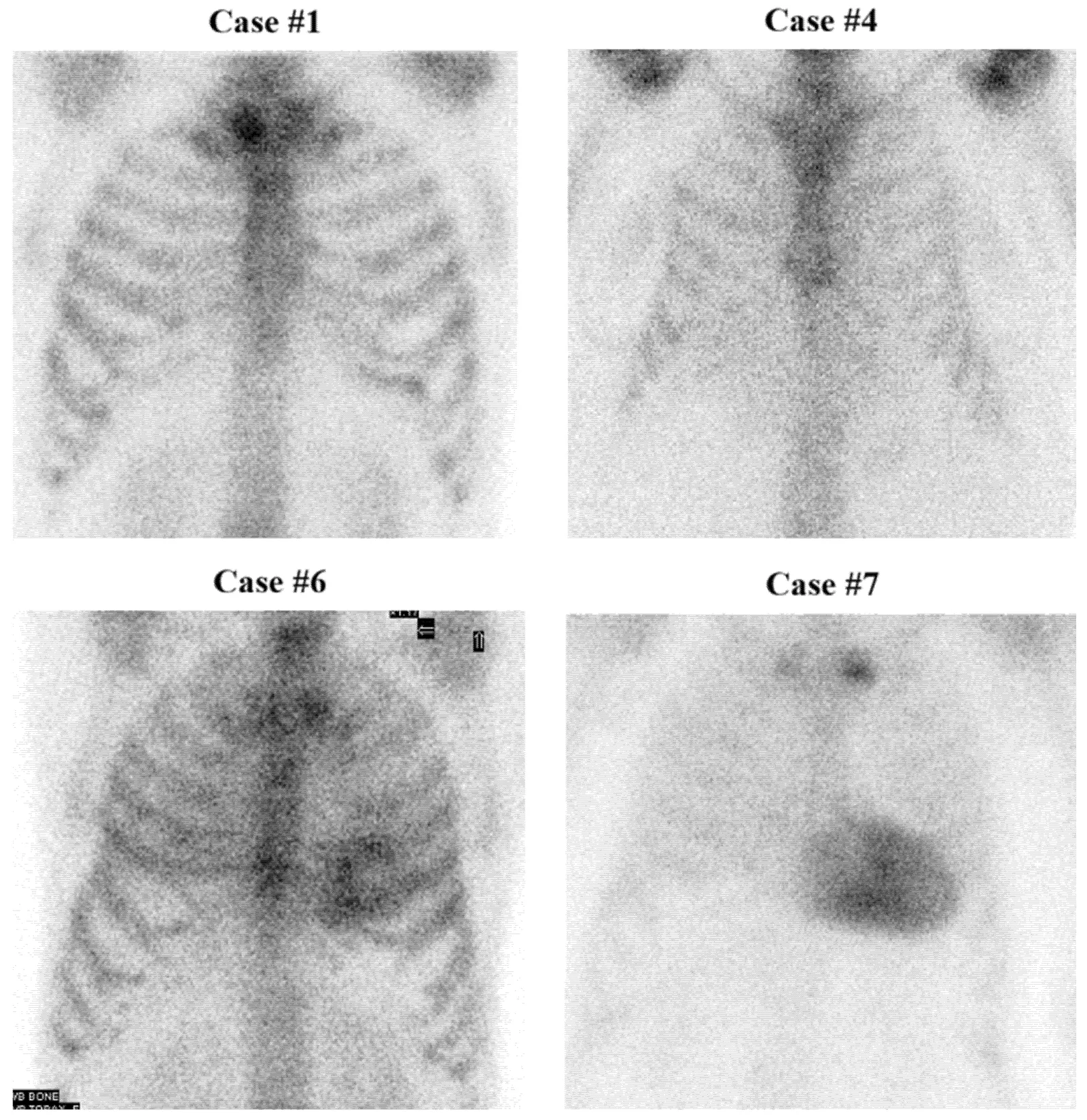
Cardiac scintigraphy with ^99m^Tc-DPD of Cases #1, #4, #6, and #7.

Overall, Case #6 showed concordant evidence of ATTR amyloidosis across tissue staining, proteomic analysis, and cardiac imaging. Case #1 had evidence of ATTR amyloid deposition in tenosynovial tissue, although cardiac scintigraphy did not support cardiac involvement. Case #4 had positive Congo red staining, but proteomic analysis did not identify increased TTR or other amyloid-associated proteins. This discrepancy likely reflects a very limited amyloid burden below the detection threshold of proteomic analysis, tissue heterogeneity, or a sampling error. Case #7 had imaging findings suspicious for ATTR-CA despite negative Congo red staining in tenosynovial tissue and absence of increased TTR in the proteomic profile. However, this patient showed increased β_2_-microglobulin (β_2_M) abundance compared with the remaining cases [Figure A1], which may warrant further evaluation for alternative amyloid subtypes. Since the tissue sample is small, it may not include areas covered by amyloid deposition, which could result in a false negative. Figure 1 shows the cardiac scintigraphy of the discussed cases.

**Figure 2:**
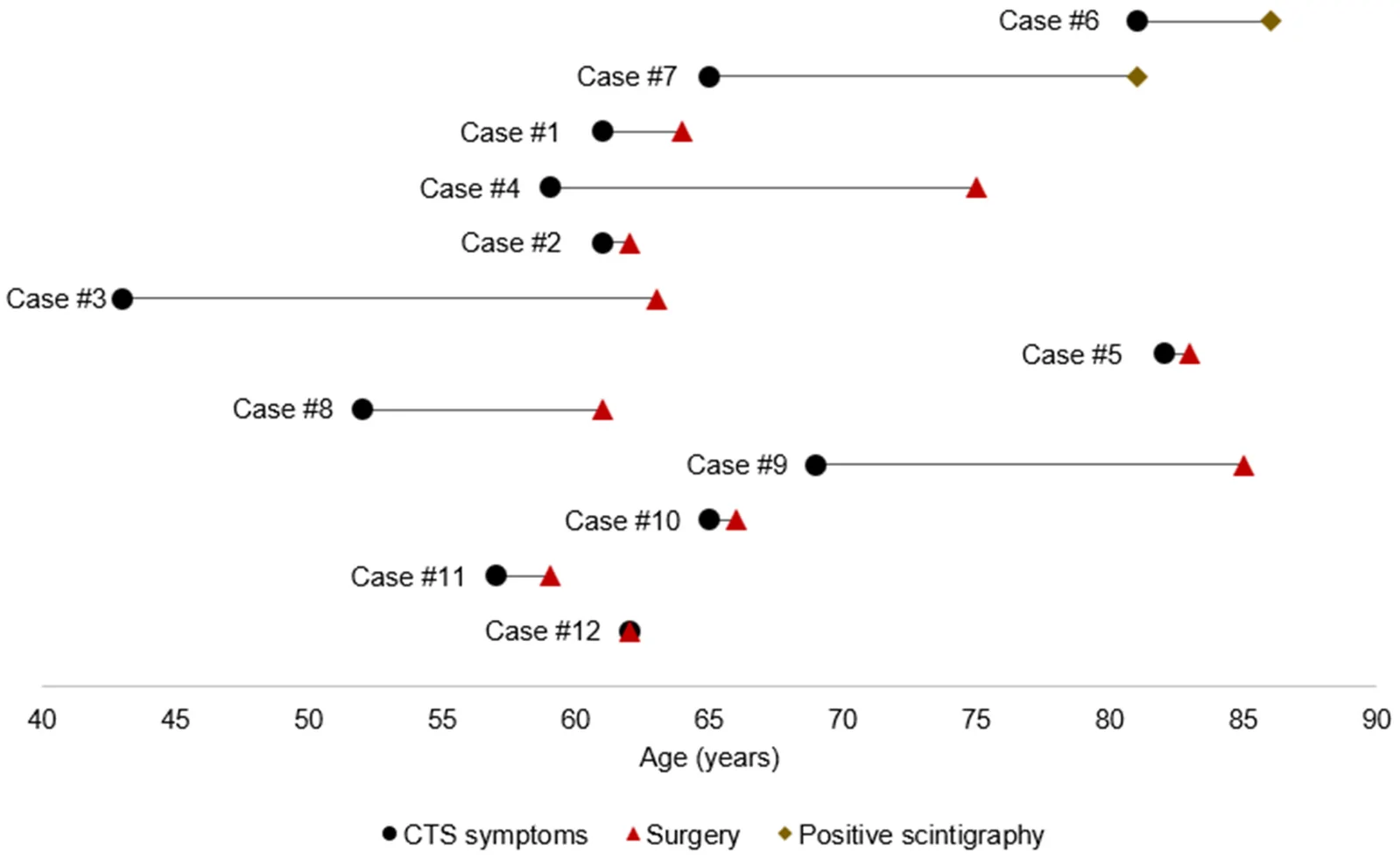
Chronological timeline illustrating the relationship between carpal tunnel syndrome (CTS) and cardiac screening results across the entire cohort. The graphic displays the diagnostic trajectory for all patients, marking the age at the onset of CTS symptoms, the age at carpal tunnel release surgery (tissue biopsy), and the moment of cardiac scintigraphy.

A comprehensive graphical representation of the temporal relationship between the onset of CTS, surgery, and cardiac screening results for all 12 patients is illustrated in Figure 2.

### 3.1. Case #1

Case #1 was a 64-year-old male with moderate bilateral CTS and a previous history of ischemic cardiomyopathy. Congo red staining and proteomic analysis were consistent with ATTR amyloid deposition in tenosynovial tissue, whereas cardiac scintigraphy did not indicate CA. The patient had low QRS voltage on ECG and reduced left ventricular ejection fraction (LVEF), initially measured at 46%. A more recent exam showed progression of cardiovascular disease, with concentric left ventricular hypertrophy (LVH) and further reduction in LVEF to 38%.

### 3.2. Case #4

Case #4 was a 75-year-old female diagnosed with mild bilateral CTS at the age of 59 years. ECG showed low QRS voltage. Although Congo red staining was positive, proteomic analysis did not show increased transthyretin abundance [Figure A1]. Moreover, no other amyloid-associated proteins were increased in this sample. These findings suggest that the Congo red result should be interpreted with caution, as it likely reflects a low-burden deposit, tissue heterogeneity, or a sampling error leading to dilution during whole-tissue proteomic analysis.

### 3.3. Case #6

Case #6 was an 86-year-old male who reported the onset of CTS symptoms at age 81 and was formally diagnosed with moderate CTS at the age of 84 years. Congo red staining and proteomic analysis were both consistent with ATTR amyloid deposition. In addition, cardiac scintigraphy showed Perugini grade 3 uptake, highly suspicious for ATTR-CA, although the absence of monoclonal protein screening precludes a definitive non-invasive diagnosis. ECG demonstrated left anterior fascicular block, and LVH was documented on both TTE and CMR. The latter also showed intramyocardial late gadolinium enhancement (LGE) in the inferolateral segment. The latter also showed intramyocardial late gadolinium enhancement (LGE) in the inferolateral segment. At the time of evaluation, the patient’s eGFR was 61 mL/min/1.73 m^2^.

### 3.4. Case #7

Case #7 was an 81-year-old male who first experienced CTS symptoms at age 65, but was only formally diagnosed with severe CTS at the age of 80 years. Cardiac scintigraphy showed Perugini grade 3 uptake, highly suspicious for ATTR-CA. However, Congo red staining of the tenosynovial biopsy was negative, and proteomic analysis did not show increased TTR abundance [Figure A1]. An exploratory analysis of the proteomic profile showed increased β2M abundance compared with the remaining patients. However, this is strictly a hypothesis-generating finding. Subsequent genetic testing, performed via polymerase chain reaction (PCR) amplification of peripheral blood DNA followed by direct sequencing of the entire coding and adjacent intronic regions, returned negative for mutations in the *B2M* gene. ECG showed first-degree atrioventricular block, and severe LVH was present on both TTE and CMR. The latest further demonstrated intramyocardial LGE in the septal segments and subendocardial late gadolinium enhancement in the basal segments, a pattern compatible with amyloid infiltration. The patient’s contemporaneous eGFR was 98 mL/min/1.73 m^2^.

## 4. Discussion

In this retrospective case series of patients undergoing tenosynovial biopsy during CTS surgery, Congo red staining identified amyloid deposits in 3 of 12 cases (25%), with 2 cases (17%) confirmed as ATTR by proteomics. Although 2 patients (17%) presented with Perugini grade 3, only one patient (8.3%) showed complete concordance across tissue histology, proteomics, and scintigraphy. These findings support the concept that CTS may represent an early or extracardiac manifestation of systemic amyloidosis, particularly ATTR. In the previous literature, the estimated prevalence of amyloid deposition in patients with CTS ranged widely from 9.3% to 34%, remaining substantially higher than in the general population [2,4,5,6,7,9,15]. For instance, while earlier studies by Nakamichi and Tachibana reported a 9.3% prevalence [5], more recent cohorts by Sekijima et al. observed rates up to 34% [2].

While Congo red staining remains a widely used screening tool, it is subject to both false-positive and false-negative results, particularly in samples with low amyloid burden or technical limitations. In this series, Case #4 initially suggested a potential false-positive Congo red result, as no corresponding increase in amyloid-associated proteins was observed in the proteomic profile. However, this discrepancy is highly nuanced. Rather than a strict false-positive, it may reflect a very low amyloid burden or a sampling error within a heterogeneous tissue sample. Furthermore, because our proteomic analysis was performed on whole tenosynovial tissue extracts rather than specifically targeting the Congo red-positive areas via laser microdissection, the localized amyloid proteomic signature may have been inadvertently diluted below the mass spectrometry detection threshold. In clinical practice, we view these diagnostic modalities as strictly complementary rather than competitive. Traditional histology serves as an accessible first-line screening tool for tissue deposition, while advanced imaging (scintigraphy, TTE, CMR) is crucial for staging the extent of functional organ involvement. However, proteomic analysis via mass spectrometry occupies the definitive tier for accurate amyloid typing. Because targeted disease-modifying therapies depend entirely on the precise identification of the amyloidogenic protein, proteomics should be pursued to resolve discordant histological results and establish a definitive tissue diagnosis within a multimodal framework.

### 4.1. Association of CTS with Cardiac Amyloidosis

As summarized in Table 3, the reported prevalence of amyloidosis in CTS cohorts varies significantly across the literature. Contemporary studies report TTR amyloid deposits in tenosynovial tissues ranging from 7.1% to 36%, while the subsequent diagnosis of cardiac amyloidosis (Perugini grade 2–3) ranges broadly from 1.0% up to 29%. Our study demonstrated a 17% rate of confirmed TTR deposits in CTS and a 17% rate of suspected ATTR-CA. While our findings align with the higher yields reported in several recent cohorts, it is crucial to note that a significant selection bias likely influences our results. Because our 12 cases were selectively sampled from a broader pool of 54 patients, the operating surgeon may have actively targeted tissues with a macroscopic appearance highly suspicious for amyloidosis.

There is scientific evidence showing that CTS is not only the most common symptom, but also an early clinical manifestation of ATTR amyloidosis (frequently ATTRwt) and should be considered a red flag [2,9,23,24]. Nakagawa et al. presented a series of patients with an antemortem diagnosis of ATTRwt amyloidosis, highlighting CTS as the most prevalent initial symptom of the disease, observed in 68% of patients [9]. Moreover, CTS preceded cardiac symptoms by 6.1 ± 4.6 years. In our cohort, two patients (16.7%) presented Perugini grade 3 uptake on cardiac scintigraphy, along with structural and tissue abnormalities on TTE and CMR, highly suspicious of ATTR-CA. However, it must be emphasised that without the formal biochemical exclusion of a monoclonal protein (via free light chains and immunofixation assays), AL amyloidosis cannot be definitively ruled out. Consequently, a positive bone scintigraphy in this setting remains highly suspicious for, rather than strictly diagnostic of, ATTR-CA, as concomitant AL amyloidosis or false-positive uptake remains a clinical possibility. In these patients, CTS symptoms preceded the suspected CA diagnosis by 5 and 16 years. There is a significant delay in the diagnosis of CA, as suggested by a previous study that estimated a delay of 22 months for all forms of CA, and an even longer duration (34 months) among patients with ATTR [25]. A one-year delay in diagnosis was associated with an increase in natriuretic peptides, cardiac conduction disturbances, and atrial fibrillation. A delay in diagnosis was negatively associated with access to treatment and long-term survival [25]. As visually summarized for the entire cohort in Figure 2, the timelines of our positive cases reinforce the concept that CTS can act as a crucial early red flag, offering a substantial window of opportunity for diagnosis before advanced cardiac involvement occurs.

Case #1 and Case #6 further demonstrate the spectrum of disease. Both showed evidence of ATTR deposition in tenosynovial tissue, but only Case #6 had concordant findings across histology, proteomics, and cardiac imaging, consistent with probable ATTR-CA. In contrast, Case #1 did not demonstrate cardiac involvement on scintigraphy, suggesting that tenosynovial amyloid deposition may precede overt cardiac disease. This observation is consistent with previous reports proposing CTS as an early clinical marker of ATTR, potentially preceding cardiac manifestations by several years.

### 4.2. Optimal Clinical Approach for an Idiopathic CTS

The optimal clinical approach to a patient with a diagnosis of idiopathic CTS remains unclear. Questions arise, such as whether obtaining tenosynovial tissue for the search of transthyretin deposits should be routinely done and whether a long-term follow-up protocol for cardiac assessments, similar to what is recommended for asymptomatic carriers of pathogenic cardiomyopathy mutations, should be considered. This consideration may be particularly relevant in patients older than 60 years and with bilateral CTS, as these features have been increasingly associated with transthyretin amyloidosis. As highlighted by Westin et al., the clinical yield for finding cardiac amyloidosis was significantly higher (>1 in 5) when focusing on nonobese men aged ≥70 years, demonstrating a strong potential for systematic screening in this specific demographic [18]. Case #7 illustrates the complementary role of multimodal assessment. Despite negative Congo red staining in tenosynovial tissue and absence of increased transthyretin in proteomic analysis, the presence of Perugini grade 3 uptake on cardiac scintigraphy, along with abnormalities on TTE and CMR, strongly supports a suspicion of CA. This discordance may reflect sampling limitations of tenosynovial biopsy or heterogeneity in amyloid deposition. The observed increase in β2M in this case was an intriguing exploratory finding, as there have been reported cases of CA related to β2M in patients not on dialysis, including families of Portuguese descent with mutations in the *B2M* gene [26,27]. However, subsequent genetic testing for this patient was negative, ruling out this hereditary form. In the absence of positive Congo red staining or confirmatory amyloid typing from cardiac tissue, this isolated proteomic signal does not establish β2M amyloidosis and highlights the necessity of correlating exploratory ‘-omics’ data with comprehensive clinical, genetic, and histological assessments.

Taken together, these findings reinforce the potential role of tenosynovial biopsy obtained during CTS surgery as an opportunity for early detection of amyloid deposition. However, the variability observed across Congo red staining, proteomic analysis, and cardiac imaging underscores the need for a multimodal diagnostic approach. Reliance on a single modality may lead to misclassification, particularly in early or low-burden disease.

## 5. Conclusions

In patients undergoing surgery for CTS, tenosynovial biopsy may reveal amyloid deposition in a subset of cases. However, accurate diagnosis and typing of amyloidosis require integration of histological, proteomic, and imaging data.

This study demonstrates that concordant findings across modalities strongly support the suspicion of ATTR-CA, whereas discordant results are not uncommon and should be interpreted with caution. Tenosynovial amyloid deposition may precede overt cardiac involvement, highlighting a potential window for early detection.

Overall, a multimodal and cautious approach is essential to avoid misclassification and to appropriately identify patients who may benefit from further evaluation for systemic amyloidosis.

## 6. Limitations

This study has several important limitations that must be explicitly acknowledged when interpreting the results. First, the small sample size and the absence of a control group limit the generalizability of our findings and preclude robust statistical inference or accurate prevalence estimation. Additionally, the retrospective nature of this specific tissue analysis introduces inherent selection and potential verification biases, as patients were included strictly based on the retrospective availability of biopsied tenosynovial tissue.

The clinical and imaging assessments were also not uniformly systematic across the cohort; transthoracic echocardiography and cardiac magnetic resonance were primarily performed in patients with positive scintigraphy, and long-term cardiac follow-up remains incomplete, limiting our ability to track disease progression. Furthermore, retrospective limitations inherently affect the uniformity of scintigraphic methodological data collection. Regarding amyloid typing and diagnosis, the lack of comprehensive laboratory screening for monoclonal gammopathy (e.g., serum and urine immunofixation, serum free light-chain assays) prevents the formal non-invasive exclusion of AL amyloidosis and the definitive non-invasive diagnosis of ATTR-CA. Similarly, the lack of systematic *TTR* genetic testing precludes the definitive differentiation between ATTRwt and variant/hereditary amyloidosis. Finally, proteomic analysis was performed on whole tissue extracts rather than laser-microdissected Congo red-positive areas. This methodological approach may dilute the amyloid proteomic signature, potentially reducing sensitivity in samples with a very low amyloid burden.

Despite these limitations, this retrospective case series highlights clinically relevant scenarios encountered in real-world practice, including discordant findings across diagnostic modalities and underscores the necessity of a multimodal and cautious approach to amyloidosis screening in patients with idiopathic CTS.

## Figures and Tables

**Table 1:** Description of the clinical, laboratory, imaging, histological, and proteomic findings of the included patients with bilateral idiopathic carpal tunnel syndrome.

Case	Gender, Age (Years)	Comorbidities	Age at Onset of CTS Symptoms	Severity CTS (EMG)—Right/Left	Congo Red Staining	Proteomic Typing	Cardiac Perugini Grade
1	M, 64	IC, Dyslipidemia	61	Moderate	1	ATTR	0
2	F, 62	Hypertension, Dyslipidemia	61	Moderate	0	Negative	0
3	F, 63	None	43	NA/ Moderate	0	Negative	0
4	F, 75	Dyslipidemia	59	Mild	1	Negative	0
5	F, 83	Hypertension	82	Severe	0	Negative	0
6	M, 86	Hypertension, Dyslipidemia	81	Moderate/Mild	1	ATTR	3
7	M, 81	Hypertension, Dyslipidemia	65	Severe	0	Negative	3
8	F, 61	None	52	Mild	0	Negative	0
9	F, 85	Hypertension	69	Moderate/Severe	0	Negative	0
10	F, 66	Hypertension, Dyslipidemia	65	Moderate/Severe	0	Negative	0
11	F, 60	None	57	Severe	0	Negative	0
12	F, 62	Dyslipidemia	62	Moderate	0	Negative	0

CTS, Carpal tunnel syndrome; EMG, Electromyography; F, Female; IC, Ischemic cardiomyopathy; M, Male; NA, Not available.

**Table 2:** Echocardiographic parameters, cardiac magnetic resonance imaging findings, and cardiac biomarker profiles of the evaluated patients.

Case	BNP (ng/L)	NT-proBNP (ng/L)	Troponin I (ng/L)	ECG Findings	IVS (mm)	PW (mm)	LV Mass (g/m^2^)	LAVI (mL/m^2^)	LVEF (%)	TAPSE (mm)	T1 (ms)	LGE Pattern
1	30	133	2.3	↓ QRS voltage	10	9	139.3	27.7	46	18.5	NP	NP
2	NP	NP	2.9	None	NP	NP	NP	NP	NP	NP	NP	NP
3	NP	NP	<1.9	↓ QRS voltage	NP	NP	NP	NP	NP	NP	NP	NP
4	NP	229	3.3	↓ QRS voltage	NP	NP	NP	NP	NP	NP	NP	NP
5	NP	NP	4.3	NP	NP	NP	NP	NP	NP	NP	NP	NP
6	NP	NP	10.5	LVH	14	NA	NA	NA	62	NA	1262	Intramyocardial inferolateral
7	NP	5882	<1.9	1st degree AVB	NA	NA	182.3	50.1	54	17	1438	Subendocardial basal and diffuse intramyocardial mid-apical
8	NP	NP	3.0	NP	NP	NP	NP	NP	NP	NP	NP	NP
9	33	135	2.8	NSTA	11	8	NA	NA	NA	NA	NP	NP
10	83	NA	<1.9	None	10	9	60.9	20.6	61	23	NA	NA
11	NP	NP	<1.9	None	NP	NP	NP	NP	NP	NP	NP	NP
12	NP	NP	2.2	NSTA	NP	NP	NP	NP	NP	NP	NP	NP

AVB, Atrioventricular block; BNP, B-type natriuretic peptide; ECG, Electrocardiogram; IVS, Interventricular septum; LAVI, Left atrial volume index; LGE, Late gadolinium enhancement; LVEF, Left ventricular ejection fraction; LV, Left ventricle; LVH, Left ventricular hypertrophy; NA, Not available; NP, Not performed; NSTA, Non-specific T-wave abnormality, NT-proBNP, N-terminal pro-B-type natriuretic peptide; PW, Posterior wall.

**Table 3:** Summary of the literature on the prevalence of tenosynovial and cardiac amyloidosis in patients undergoing carpal tunnel release.

Study (Year)	Total Patients (n)	Mean/Median Age (Years)	Male Sex (n, %)	TTR Amyloid Deposits in CTS (n, %)	Cardiac Amyloidosis (Perugini 2–3) (n, %)	AL Amyloidosis Excluded
Sperry et al. (2018) [7]	98	68	51 (52%)	7 (7.1%)	1 (1.0%)	Yes
Zegri-Reiriz et al. (2019) [16]	101	69.5	32 (32%)	NP	2 (2.0%)	Yes
Sugiura et al. (2021) [17]	79	71.6	32 (41%)	27 (34%)	3/16 (19%)	Yes
Westin et al. (2022) [18]	250	70.4	125 (50%)	NP	12 (4.8%)	Yes
Ladefoged et al. (2022) [19]	120	74.5	109 (91%)	NP	10/67 (15%)	Yes
Yamada et al. (2023) [20]	33	69.8	6 (18%)	12 (36%)	2/7 (29%)	No
Takashio et al. (2023) [21]	700	63.9	253 (36%)	116 (17%)	6/120 (5.0%)	Yes
Razvi et al. (2026) [22]	555	68.7	240 (43%)	174 (31%)	32/116 (28%)	Yes
Martins et al. (2026) (This study)	12	65	3 (25%)	2 (17%)	2 (17%)	No

AL, Amyloid light-chain; NP, Not performed; TTR, Transthyretin.

## Data Availability

The data presented in this article are not readily available because they are part of an ongoing study. Requests for access to these data should be made to the corresponding author, Elisabete Martins, ebernardes@med.up.pt.

## Supplementary Materials

The following supporting information can be downloaded at: https://www.mdpi.com/article/10.3390/jcm15176543/s1, Table S1: Quantitative proteomic profiling of amyloidogenic and canonical amyloid-signature proteins in tenosynovial tissue samples. The table details the protein name, gene symbol, UniProt accession number, total number of unique peptides, and overall sequence coverage (%) for each identified protein. The normalized abundance values are provided for each of the 12 patient samples (F1–F12). Empty cells represent abundance values below the detection threshold for that specific sample.

## Abbreviations

The following abbreviations are used in this manuscript:

ATTR	Transthyretin-related amyloidosis
ATTR-CA	Transthyretin cardiac amyloidosis
ATTRwt	Transthyretin-related amyloidosis wild type
AVB	Atrioventricular block
CA	Cardiac amyloidosis
CMR	Cardiac magnetic resonance
CTS	Carpal tunnel syndrome
ECG	Electrocardiogram
EMG	Electromyography
IC	Ischemic cardiomyopathy
LAFB	Left anterior fascicular block
LGE	Late gadolinium enhancement
LVEF	Left ventricular ejection fraction
LVH	Left ventricular hypertrophy
Tc-DPD	Technetium 3,3-diphosphono-1,2-propanodicarboxylic acid
TTE	Transthoracic echocardiogram
Β2M	β2-microglobulin

## Appendix A

Proteomic profiling of tenosynovial tissue demonstrated heterogeneous expression patterns among patients with CTS (Figure A1). Case #1 and #6 demonstrated increased transthyretin expression together with elevated apolipoproteins, consistent with the proteomic profile of ATTR. In contrast, Case #4, despite positive Congo red staining, did not demonstrate a clear increase in transthyretin or other canonical amyloid-associated proteins, a low amyloid burden, tissue heterogeneity, or sampling error. Case #7 showed a distinct proteomic pattern characterized by elevated β2M despite the absence of increased transthyretin expression; however, this remains an exploratory finding of uncertain clinical significance, as subsequent genetic testing was negative for *B2M* mutations. Overall, the proteomic analysis highlighted substantial inter-patient variability and reinforced the importance of integrating histological, proteomic, and imaging findings for the interpretation of amyloid deposition in CTS patients.
